# Notes and News

**Published:** 1898-03-26

**Authors:** 


### Notes and News.

(Collected and Communicated.)

HOSPITAL MEETINGS.

The Duke of Cambridge has consented to preside at the Festival Dinner in aid of the funds of the Royal London Ophthalmic Hospital, at the Grand Hotel, Charing Cross, on Friday, May 6th.

London Homœopathic Hospital.—The Annual General Meeting, Thursday, March 24th, 1898, 4 o'clock, at the Hospital.

H.R.H. the Duke of Cambridge has promised to be present at the dinner in aid of the London Hospital at the Mansion House on May 18th.

A festival dinner in aid of the Chelsea Hospital for Women will be held at the Hotel Metropole on June 16th, at which Lord Glenesk will preside.

The annual dinner of the Royal Hospital for Incurables, Putney Heath, will take place on June 7th, at the Hotel Metropole. Alderman Truscott will preside.

The Duke of Cambridge has promised to preside at the festival dinner in aid of the funds of the Royal London Ophthalmic Hospital at the Grand Hotel, Charing Cross, on May 6th.

H.R.H. the Duke of Connaught, president of the City of London Hospital for Diseases of the Chest, has promised to take the chair at the festival dinner to be held in connection with the institution at the Hotel Cecil on May 12th.

According to the *British Medical Journal* it may safely be said that some 50,000 copies of "Quain's Dictionary of Medicine" are in the hands of the profession. This is interesting, but can it be true? "Churchill's Medical Directory" gives the total number of persons possessing British qualifications as 34,903, and we doubt if a quarter of these have a Quain upon their shelves. Where, then, are the 50,000? The answer is, partly in the colonies and America, but largely in the hands of the public. No book on medicine has been more consulted by the public than "Quain's Dictionary," notwithstanding the fulminations issued against medical writing for public use; fulminations, by the bye, which are apt to be hurled with but gentle vigour against those highly placed in the profession.

A balance of £20 remains from the subscriptions for Viscount Peel's portrait, and it is proposed to hand over this sum to the Westminster Hospital.

The fifty-third annual dinner of the German Hospital will be held at the Whitehall Rooms on April 15th. The treasurer, Baron von Schröder, has promised to preside.

A performance in aid of the Prince of Wales's Hospital Fund is to be given by the members of the Savage Club at Her Majesty's Theatre. Mrs. Tree and Sir Henry Irving have both promised their services.

A very successful performance was given on the 23rd at the Eden Theatre, at Cannes, in aid of the British Hospital there. The Prince of Wales and the Duches of Albany were patrons of the event.

Measles still continues to be prevalent in London but the deaths from that cause fell somewhat last week, compared with the week before, the numbers for the last four weeks being 106, 115, 141, and 134 respectively. Compared, however, with some provincial towns London's death-rate from measles is trivial, the rate per 1,000 being 1·6 in London, 2 6 in Bristol, 3·1 in Huddersfield, 3·2 in Oldham, 3·4 in Brighton, and 4·0 in Leicester.

The new *concierge* of the Pasteur Institute in Paris has an interesting history, in that he was the first patient who was treated by Pasteur with his hydrophobia prophylactic, then quite new, while acting as shepherd. He had been badly bitten by a mad dog, and submitted cheerfully to the untried treatment. In recognition, Pasteur presented him with a silver medal, employed him in his science laboratories, and he has just been appointed *concierge* of the institute.

A sudden dread seems to have seized upon certain medical men lest, if the Midwives Bill should happen to be passed, the midwives so established by law should assume the title of licentiate in midwifery, and dub themselves L M. It no doubt would be very wrong, but it seems to us that it is exactly what they would be most likely to do. They clearly must call themselves by some name or other, and it is hardly likely that, even to please the British Medical Association, they will call themselves obstetric nurses, and if they are not to be midwives what are they to be?

The death of Dr. Charles West, which occurred in Paris last Saturday after a few days' illness, removes from the ranks of our profession one who, years ago, was a very prominent figure in medical circles. Dr. West was born in 1816, and graduated at the University of Berlin in 1837, becoming a member of the Royal College of Physicians in 1842. In 1849, in conjunction with the late Dr. Bence Jones, he succeeded in founding the Hospital for Sick Children, Great Ormond Street. He held many high offices, and wrote many books, but there is no doubt that the work by which he was best known was his "Lectures on the Diseases of Infancy and Childhood," a book which was for many years the great work of reference on those subjects.

